# Zika virus RNA excretion in sweat with concomitant detection in other body fluid specimens

**DOI:** 10.1590/0074-02760200339

**Published:** 2021-01-25

**Authors:** Armando Menezes-Neto, Marcia da Costa Castilho, Guilherme Amaral Calvet, Edna Oliveira Kara, Camila Helena Aguiar Bôtto-Menezes, Marcus Vinícius Guimarães Lacerda, Gerson Fernando Mendes Pereira, Silvana Pereira Giozza, Ximena Pamela Diaz Bermudez, Noemia Santana Lima, Kayvon Modjarrad, Nathalie Broutet, Ana Maria Bispo de Filippis, Rafael Freitas Oliveira Franca, Study Team ZIKABRA

**Affiliations:** 1 Fundação Oswaldo Cruz-Fiocruz, Instituto Aggeu Magalhães, Recife, PE, Brasil; 2 Fundação de Medicina Tropical Doutor Heitor Vieira Dourado, Manaus, AM, Brasil; 3 Instituto Nacional de Infectologia Evandro Chagas, Rio de Janeiro, RJ, Brasil; 4 World Health Organization, Geneva, Switzerland; 5 Universidade do Estado do Amazonas, Manaus, AM, Brasil; 6 Fundação Oswaldo Cruz-Fiocruz, Manaus, AM, Brasil; 7 Ministério da Saúde, Secretaria de Vigilância em Saúde, Departamento de Doenças de Condições Crônicas e Infecções Sexualmente Transmissíveis, Brasília, DF, Brasil; 8 Organização Pan Americana de Saúde/Organização Mundial da Saúde, Universidade de Brasília, Departamento de Saúde Pública, Brasília, DF, Brasil; 9 Henry M Jackson Foundation for the Advancement of Military Medicine, Bethesda, MD, USA; 10 Walter Reed Army Institute of Research, Silver Spring, Maryland, USA; 11 Fundação Oswaldo Cruz-Fiocruz, Instituto Oswaldo Cruz, Rio de Janeiro, RJ, Brasil

**Keywords:** Zika virus, diagnosis, persistence

## Abstract

We evaluated sweat, blood and urine specimens obtained from an ongoing cohort study in Brazil. Samples were collected at pre-established intervals after the initial rash presentation and tested for Zika virus (ZIKV) RNA presence by real-time reverse transcriptase polymerase chain reaction (rRT-PCR). From 254 participants with confirmed infection, ZIKV RNA was detected in the sweat of 46 individuals (18.1%). Sweat presented a median cycle threshold (Ct) of 34.74 [interquartile range (IQR) 33.44-36.04], comparable to plasma (Ct 35.96 - IQR 33.29-36.69) and higher than urine (Ct 30.78 - IQR 28.72-33.22). Concomitant detection with other specimens was observed in 33 (72%) of 46 participants who had a positive result in sweat. These findings represent an unusual and not yet investigated virus shedding through eccrine glands.

Zika virus (ZIKV) is a recently re-emerged arbovirus. In humans, ZIKV infection is characterized by a febrile or exanthematous illness, and has been linked to a series of congenital defects in children collectively denominated Congenital Zika Syndrome (CZS) and neurologic manifestations in adults, such as Guillain-Barre Syndrome.^1^ ^,^ ^2^ ZIKV laboratory detection is usually performed by real time reverse transcriptase polymerase chain reaction assays (rRT-PCR), most often from blood samples (serum or plasma), whose sensitivity is highest up to five days after symptoms onset.^3^ Several reports have described the presence of ZIKV RNA in other body fluids than blood, such as urine,^4^ semen,^5^ saliva,^6^ vaginal^7^ and rectal secretions^8^ with variable sensitivity.^9^ Overall, ZIKV molecular detection differs according to the days of symptoms (from the initial rash report) and the type of specimen analyzed, besides intrinsic methodology variations. Nowadays it is well accepted that ZIKV persists for longer in semen when compared to other body fluids, with reported duration of up to six months after disease onset.^10^ Clearly, these findings impact directly on virus transmission (e.g., routes of infection) and diagnosis, where specific specimens are preferable to others according to the days of symptoms.^10^ Hence, it is important to understand ZIKV kinetics in human body fluids to best advice on timeframes as well as alternative samples for diagnosis. Thus, improved knowledge about the virus biology (i.e., types of tissues and cells infected, and its kinetics) may impact on preventive transmission measures. Here we report the detection of ZIKV RNA in sweat samples from 46 of 254 individuals with confirmed ZIKV infection recruited from an ongoing cohort study in Brazil. In our cohort, ZIKV persistence was assessed by rRT-PCR in different body fluids from individuals with confirmed infection through a multicenter prospective study (the ZIKABRA Study). Initially, at the screening visit, ZIKV acute infection was confirmed by processing only blood (plasma) and urine samples from individuals with rash presentation or previous rash history and their symptomatic and/or asymptomatic household/sexual contacts. Participants with a positive rRT-PCR on plasma and/or urine at the screening visit had other fluid specimens collected (blood, urine, semen, vaginal secretion/menstrual blood, rectal swab, and sweat) at different intervals after the initial rash documentation (as specified on figure legends), samples were further tested for ZIKV RNA presence by rRT-PCR. From June 2017 to June 2019, ZIKV infection was confirmed in 254 participants recruited in Manaus, northern Brazil. From these, 223 (87.8%) participants were symptomatic (index) cases and 31 (12.2%) were asymptomatic household/sexual contact subjects. Since it is an ongoing cohort study, here we describe the findings of ZIKV RNA presence in sweat samples and its comparison to laboratory gold standard specimens only (blood and urine), detection of ZIKV RNA in rectal swab samples was previously published,^8^ other body fluids specific findings will be published elsewhere. For this study, we collected sweat by gently rotating a sterile cotton swab on participants’ forehead. Swabs were further immersed in 1 mL of sterile Hank’s Balanced Salt Solution - HBSS (ThermoFisher Scientific, https://www.thermofisher.com) and stored at −80ºC. ZIKV detection was performed by processing 300 μL of each specimen for RNA extraction in an automated nucleic acid purification platform with the Maxwell 16 Viral Total Nucleic Acid Purification kit (Promega Corporation, https://www.promega.com), in a final elution volume of 70 μL. rRT-PCR reactions were performed with a commercially available kit from Instituto de Tecnologia em Imunobiológicos Biomanguinhos, approved by Agência Nacional de Vigilância Sanitária/ANVISA, Brazil (registry no. 80142170032; https://www.bio.fiocruz.br) on a QuantStudio 5 Real-Time PCR system (ThermoFisher Scientific, https://www.thermofisher.com). Samples were considered positive when the target amplification was detected within 38 amplification cycles, followed by positive detection of an internal control reaction (an RNA virus-like particle individually added to each specimen before RNA extraction). Detailed clinical data collection, sampling and laboratory protocol description for this study are described by Calvet et al.^11^ The study was approved by Research Ethics Review Committee (WHO ERC), Protocol ID: ERC.0002786; Brazilian National Research Ethics Commission (CONEP) (CAAE: 62,518,016.6.1001.0008); Institutional Ethics and Research Committee of the Evandro Chagas National Institute of Infectious Diseases, Fiocruz, Rio de Janeiro (CAAE: 62,518,016.6.2002.5262), Ethics and Research Committee of the Rio de Janeiro’s Municipal Secretary of Health (CAAE: 2,518,016.6.3001.5279); Institutional Ethics and Research Committee of the Institute Aggeu Magalhães, Fiocruz, Recife (CAAE: 62,518,016.6.2001.5190) and Institutional Ethics and Research Committee of the Tropical Medicine Foundation, Manaus, Amazonas (CAAE: 62,518,016.6.2003.0005). Informed consent was obtained from all participants prior to enrollment. Sweat samples from all 254 subjects were analyzed at a maximum of five time points (here defined as “study visit”) after the initial rash presentation and/or ZIKV infection confirmation (specific for asymptomatic household/sexual contacts). Each study visit has a scheduled maximum and minimum collection window. For this manuscript, the day of sample collection was adjusted to correspond to the exact number of days after the initial rash report. Positive rRT-PCR results for ZIKV RNA in sweat were found in 46 (18.1%) of 254 participants, of which five participants returned a positive result in more than one study visit analyzed. From the 46 positive sweat participants, a total of 45 presented symptomatic infection, where rash was the most reported symptom, followed by itchiness and fever, no other unusual symptoms were reported (Table). No other comorbidities were documented, other common chronic infectious diseases (human immunodeficiency virus, Hepatitis C, Hepatitis B, and syphilis) were also tested and returned negative. A single participant was defined as an asymptomatic household contact (participant 12, Fig. 1A).

**TABLE:** Participants characteristics and clinical data reported at screening visit (defined as rash presentation or Zika virus (ZIKV) initial positive detection for household/sexual asymptomatic subjects). Clinical data was collected at outpatients study clinic, only those participants with a positive detection at sweat are presented

Participants	Total 46	Age (mean ± SD)
Male	16	30 (± 10.9)
Female	30	43 (± 12.2)
Symptoms reported at screening visit		
Rash		97.8% (45/46)
Fever		84.7% (39/46)
Itchiness		91.3% (42/46)
Conjunctival hyperemia		78.2% (36/46)
Arthralgia		80.4% (37/46)
Periarticular edema		67.4% (31/46)

SD: standard deviation.

**Fig. 1:**
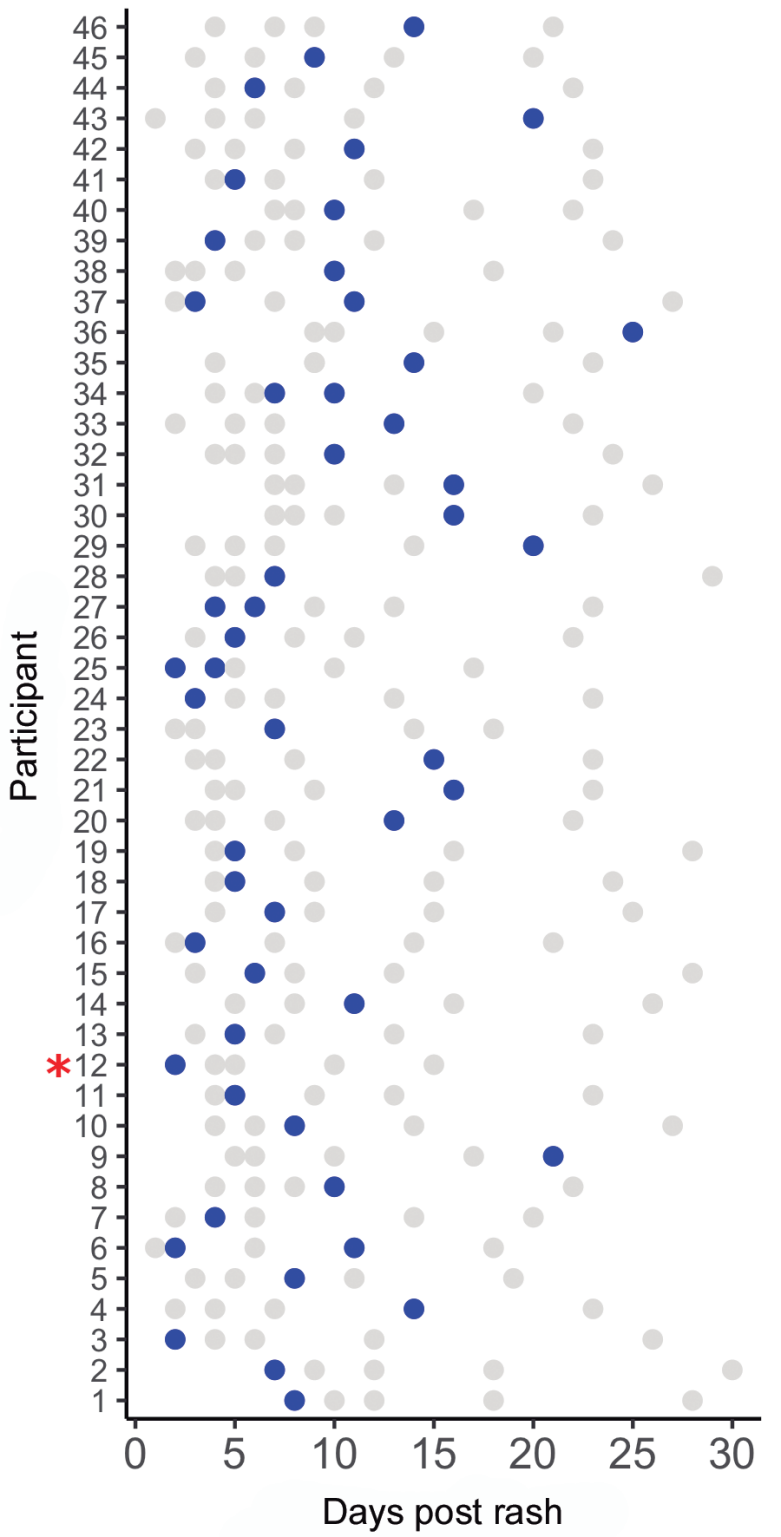
(A) Zika virus (ZIKV) infection confirmation by real time reverse transcriptase polymerase chain reaction (rRT-PCR). ZIKV RNA detection in sweat samples from 46 participants of 254 confirmed infection cases in Manaus, northern Brazil, analyzed at different time points (according to days of rash report). Detections in sweat at two distinct time points were reported for five participants: (participant IDs 6, 25, 27, 34, and 37). A single participant was defined as asymptomatic household contact (participant 12 - asterisk). Blue dots - ZIKV detection in sweat. Gray dots - detection at other body fluid (blood, urine, or semen, not specified on the figure) according to days post rash.

So far in our cohort, the longest observed duration of ZIKV RNA presence in sweat was 25 days after the initial rash report. Overall, the frequency of positive samples decreased over-time, where most of the detections were observed within 10 days after the initial rash report, for all samples analyzed (Fig. 2A). Sweat samples presented a cycle threshold (Ct) median value of 34.74 [interquartile range (IQR) 33.44-36.04], slightly lower (not significant) than plasma (Ct 35.96 - IQR 33.29-36.69), urine presented a Ct median value of 30.78 (IQR 28.72-33.22), suggesting the presence of a higher viral load. By comparing the median Ct among plasma and urine (from the 46 sweat positive participants only), sweat median Ct was closest to plasma, the most employed specimen for ZIKV diagnosis (Fig. 2A inset). Concomitant detection (defined as a positive sweat rRT-PCR result paired to another body fluid specimen at the same time point) was observed in 33 (72%) of 46 participants, of which 22 (48%) returned positive results in more than two specimens. Urine was the most frequent co-detected specimen (20/46), followed by saliva (8/46), semen (5/16), rectal swab (5/46), vaginal swab (4/30) and plasma (4/46) (Fig. 2B). Here, detection of Zika virus RNA in human swab sweat samples demonstrates the presence of virus RNA in the excretory system of naturally infected patients. Reports on virus shedding in sweat are scarce, however, RNA from another flavivirus, Hepatitis C virus (HCV), was detected in sweat samples from chronic-infected patients,^12^ supporting the idea of virus shedding in the excretory system, however, whether HCV particles in sweat are truly infectious remains elusive. In adults, sweat rates can reach 2-4 liters per hour or 10-14 liters per day,^13^ thus it is not surprising to find viral RNA being eliminated through sweat. We cannot discard the potential virus replication in sweat glands (as reported for HCV), although the ability of eccrine gland cells to express ZIKV entry receptors TIM (T-cell immunoglobulin and mucin domain), and TAM (Tyro3, Axl, and Mer) remains unknown. Cell discamation was unlikely to interfere in our results since samples were collected from visible droplets employing a soft cotton head swab. In addition, the epidermis is constituted by highly keratinized cells containing no blood vessels which are unlikely to support virus replication. Unlike the study with HCV chronic infected patients whose sweat was artificially induced employing a combination of chemicals and an electric stimulus at the forearm,^12^ sweat droplets collected by our study were naturally produced (reflecting a natural body adaptation to high temperatures - a common feature in tropical areas) and collected from patients without any special preparation.

**Fig. 2:**
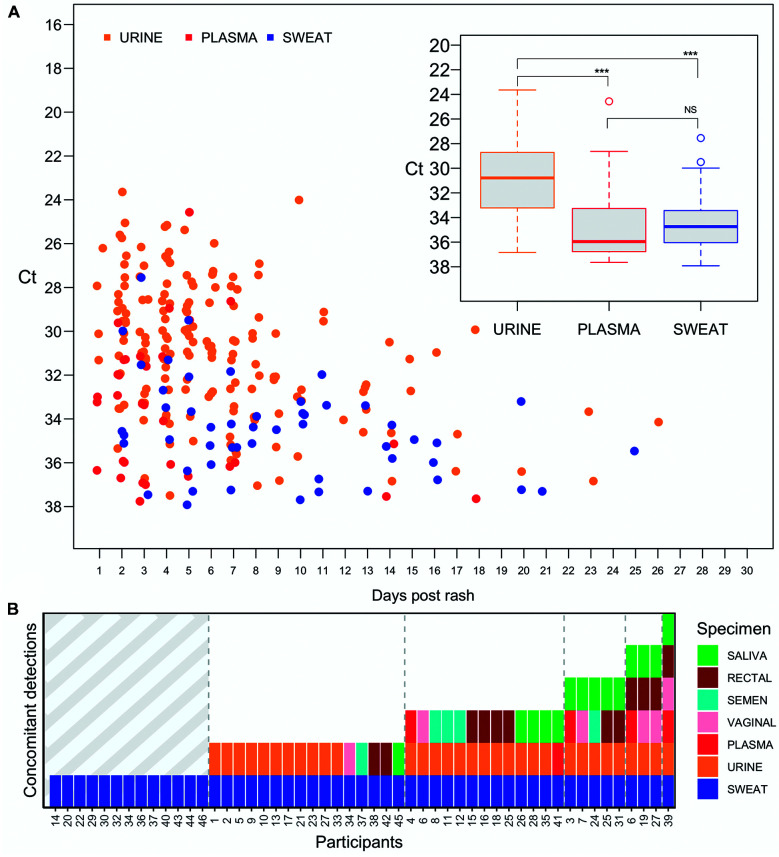
(A) Zika virus (ZIKV) RNA detection by real time reverse transcriptase polymerase chain reaction (rRT-PCR) according to the days of symptoms onset (days post rash report - “x” axis), Cycle threshold (Ct) rRT-PCR value (“y”axis) and body fluid specimen (urine, plasma and sweat) (figure legend colors). (inset) Median Ct value from 46 participants with positive rRT-PCR result in urine, plasma, and sweat body fluid specimens. Cut-off values were set above Ct 38 on rRT-PCR result. Ct - cycle threshold. NS: not significant; ***p < 0.001 analyzed by One-way ANOVA. (B) ZIKV RNA concomitant detection according to the body fluid specimen analyzed. rRT-PCR concomitant detection (“y” axis) refers to an individual positive detection on that specimens from the same participant (as specified on figure legend) according to its study number identification.

Urine was the most co-detected specimen, suggesting that ZIKV shedding through the excretory system is a common finding. Albeit the presence of ZIKV infectious particles in sweat remains unknown, transmission by direct contact (i.e., by touching an infected individual) is unlikely to occur since the skin is a natural barrier not permissive to ZIKV replication. On the other hand, a previous study has reported a possible transmission by contact with tears^14^ and ZIKV RNA has been detected in conjunctival,^15^ rectal^8^ and vaginal^16^ swabs, suggesting a possible transmission through mucosal tissues. All patients here were confirmed to be ZIKV infected through rRT-PCR performed from blood and/or urine after the rash presentation. Sweat detection was always associated with detection in other specimens even in the cases of single detection in sweat (14 participants) as these had been previously screened and confirmed to be ZIKV infected through blood and/or urine samples. Importantly, specimens were randomly processed for RNA extraction and rRT-PCR reaction, decreasing the chances of sample cross contamination given that for the same patient specimens were independently analyzed. Median Ct in sweat was comparable to plasma (albeit slightly slower), the most employed specimen for ZIKV diagnosis, however, the frequency of ZIKV RNA presence in sweat seems to be lower than blood (the gold standard specimen for laboratory diagnosis). The frequency of ZIKV RNA detection in sweat was 18.1% (46/254), considering those patients with a positive result during the acute phase of infection only (up to 20 days after symptoms onset). As expected, the frequency of positive samples decreased over time, except for semen that remains positive for longer as previously reported.^9^ ^,^ ^17^ Intriguingly, ZIKV detection in sweat was more frequently associated with its detection in urine, this may be explained by the fact that the median duration of ZIKV viremia in serum is five days^18^ whereas in urine ZIKV remains detectable above 10 days after disease onset.^4^ In our findings, ZIKV RNA in sweat was also detected at later time points, suggesting a prolonged time virus persistence in the excretory system.
